# Reliability of three-dimensional color flow Doppler and two-dimensional pulse wave Doppler transthoracic echocardiography for estimating cardiac output after cardiac surgery

**DOI:** 10.1186/s12947-019-0155-1

**Published:** 2019-04-03

**Authors:** Guang-wei Hao, Yang Liu, Guo-guang Ma, Jun-yi Hou, Du-ming Zhu, Lan Liu, Ying Zhang, Hua Liu, Ya-min Zhuang, Zhe Luo, Guo-wei Tu, Xiao-mei Yang, Hai-yan Chen

**Affiliations:** 1 Department of Critical Care Medicine, Zhongshan Hospital, Fudan University, No. 180 Fenglin Road, Xuhui District, Shanghai, 200032 People’s Republic of China; 2 Department of Echocardiography, Zhongshan Hospital, Fudan University, No. 180 Fenglin Road, Xuhui District, Shanghai, 200032 People’s Republic of China

**Keywords:** Three-dimensional color flow doppler, Two-dimensional pulse wave doppler, Cardiac output, Cardiac surgery

## Abstract

**Background:**

Three-dimensional color flow Doppler (3DCF) is a new convenient technique for cardiac output (CO) measurement. However, to date, no one has evaluated the accuracy of 3DCF echocardiography for CO measurement after cardiac surgery. Therefore, this single-center, prospective study was designed to evaluate the reliability of three-dimensional color flow and two-dimensional pulse wave Doppler (2D-PWD) transthoracic echocardiography for estimating cardiac output after cardiac surgery.

**Methods:**

Post-cardiac surgical patients with a good acoustic window and a low dose or no dose of vasoactive drugs (norepinephrine < 0.05 μg/kg/min) were enrolled for CO estimation. Three different methods (third generation FloTrac/Vigileo™ [FT/V] system as the reference method, 3DCF, and 2D-PWD) were used to estimate CO before and after interventions (baseline, after volume expansion, and after a dobutamine test).

**Results:**

A total of 20 patients were enrolled in this study, and 59 pairs of CO measurements were collected (one pair was not included because of increasing drainage after the dobutamine test). Pearson’s coefficients were 0.260 between the CO-FT/V and CO-PWD measurements and 0.729 between the CO-FT/V and CO-3DCF measurements. Bland-Altman analysis showed the bias between the absolute values of CO-FT/V and CO-PWD measurements was − 0.6 L/min with limits of agreement between − 3.3 L/min and 2.2 L/min, with a percentage error (PE) of 61.3%. The bias between CO-FT/V and CO-3DCF was − 0.14 L/min with limits of agreement between − 1.42 L /min and 1.14 L/min, with a PE of 29.9%. Four-quadrant plot analysis showed the concordance rate between ΔCO-PWD and ΔCO-3FT/V was 93.3%.

**Conclusions:**

In a comparison with the FT/V system, 3DCF transthoracic echocardiography could accurately estimate CO in post-cardiac surgical patients, and the two methods could be considered interchangeable. Although 2D-PWD echocardiography was not as accurate as the 3D technique, its ability to track directional changes was reliable.

## Background

Hemodynamic optimization has been shown to improve postoperative outcomes for moderate- and high-risk patients [1] and for cardiac surgical patients [2]. Cardiac output (CO) monitoring plays an important role in hemodynamic optimization, and the gold standard method for CO measurement is thermodilution through a pulmonary artery catheter (PAC) [3]. However, this method is invasive, expensive, and time-consuming [4], and the effectiveness of this device in cardiac surgical patients is controversial [5]. A FloTrac/Vigileo™ (FT/V) system is a less invasive technique for CO monitoring than PAC, and the reliability of the third generation FT/V system for CO measurement is excellent in patients with normal systemic vascular resistance [6, 7].

Echocardiography is a noninvasive, convenient platform for measuring CO. [8] It is very useful in hemodynamic monitoring and is recommended by experts to direct fluid management of patients in shock [9]. However, current evidence does not prove its accuracy, or even its ability to track directional changes. Moreover, most previous studies mainly focused on the accuracy of two-dimensional (2D) techniques [10]. Three-dimensional (3D) techniques for CO evaluation should be more accurate than 2D ones because 3D echocardiography can more accurately evaluate the aortic root and aortic valve area [11–16]. 3D color flow Doppler (3DCF) is a new technique that is more convenient than traditional 3D echocardiography because it does not require reconstruction. However, to date, no one has evaluated the accuracy of 3DCF echocardiography for CO measurement after cardiac surgery. Therefore, this single-center, prospective study was designed to evaluate the agreement between CO measured by the third generation FloTrac/Vigileo™ system as a reference method and CO measured by 3DCF and 2D echocardiography in post-cardiac surgical, hemodynamically stable patients.

## Methods

This study was approved by the Ethics Committee of Zhongshan Hospital (Shanghai, China), which is affiliated with Fudan University (No. B2017–139). Informed consent was obtained from all study participants.

### Patient selection

This single center, prospective study was conducted in adult cardiac surgical patients at the Zhongshan Hospital of Fudan University from 1 May to 31 May, 2018. In our hospital, all cardiac surgical patients were routinely transferred to the cardiac surgical intensive care unit with tracheal intubation after surgery. After admission, research personnel screened all patients for enrollment. The inclusion criteria were: 1) cardiac surgical patient; 2) good acoustic window; and 3) low dose or no dose of vasoactive drugs. The exclusion criteria were: 1) age < 18 and > 80 years; 2) aortic valve disease; 3) arrhythmias, extrasystole included; and 4) preoperative ejection fraction < 0.4.

Enrolled patients were sedated via propofol and remifentanil for the duration of CO measurements. No inspiratory efforts were observed and no muscular blocking agents were used in this study. Patients were ventilated using intermittent positive pressure ventilation. The ventilatory parameters were set up as follows: tidal volume: 6–8 mL/kg predicted body weight; positive end-expiratory pressure: 5 cmH_2_O; respiratory rate: 12–16 breaths/minute; fraction of inspired oxygen 50–100%. These parameters were adjusted to meet the goal of PaCO_2_ ≤ 45 mmHg and oxygen saturation (SaO_2_) > 96%. Both the dose of sedatives and the parameters of ventilation were unchanged in the duration of this study.

### Measurements

**Fig. 1:**
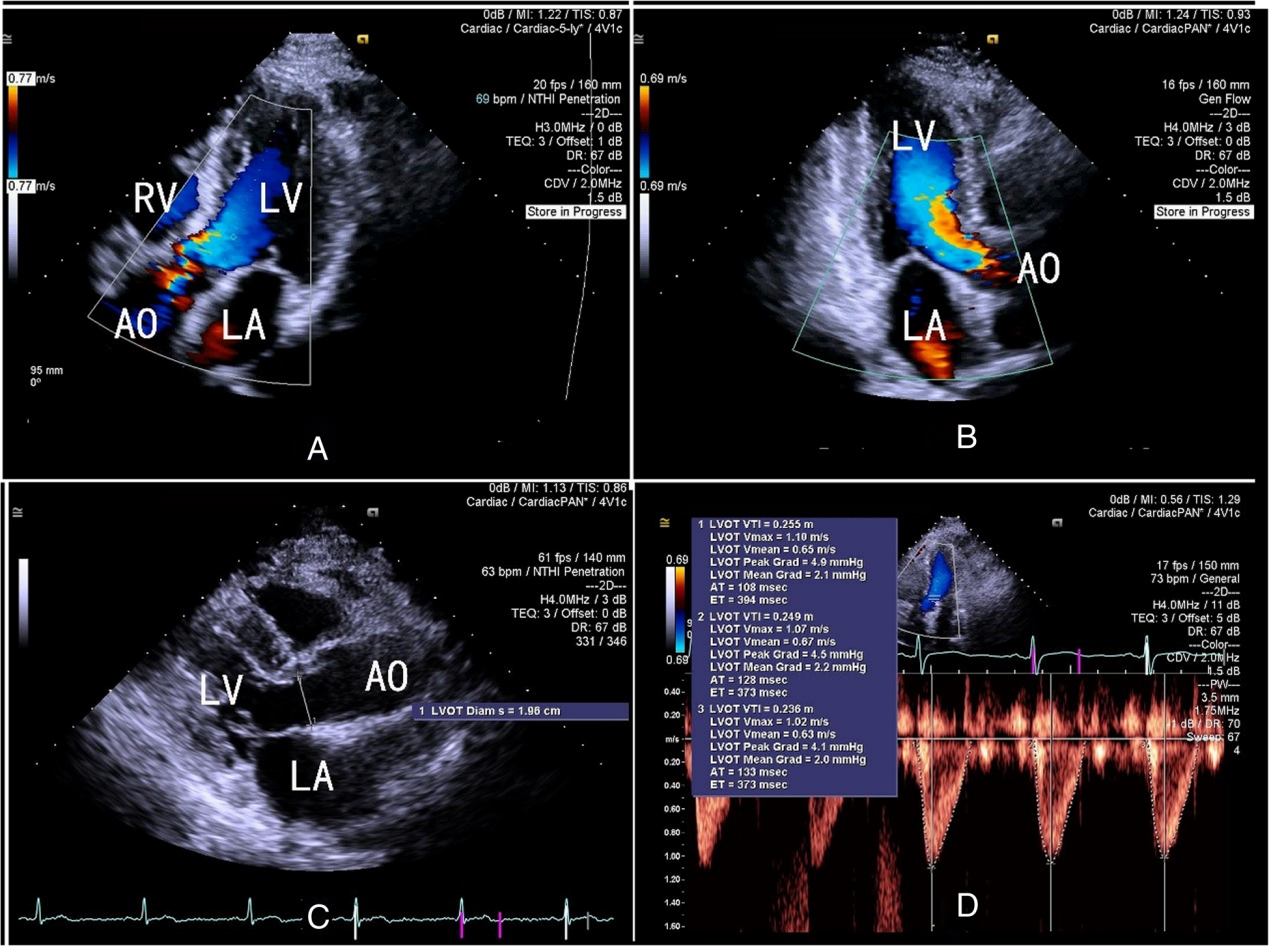
CO assessment using 2D-PWD transthoracic echocardiography. Cardiac output was calculated using the LVOT diameter, LVOT VTI, and heart rate. In routine practice, LVOT velocity–time integrals (VTIs) could be acquired in the apical five chamber view (panel **a**) or apical long axis view (panel **b**). Diameters of the left ventricular outflow tract (LVOT) were measured in the parasternal left ventricular long axis view during the early phase of ventricular systole (panel **c**). LVOT VTI was automatically measured for three consecutive beats and averaged (panel **d**). RV, right ventricle; LV, left ventricle; LA, left atrium; AO, aorta

After the intensivists and echocardiography experts confirmed the enrollment, the FloTrac pressure transducer was connected to the radial artery catheter of each patient and the Vigileo™ monitor (third version). 2D images were acquired using the Siemens Acuson SC2000™ Prime ultrasound system with a 4V1c transducer (1.25–4.5 MHz), as shown in Fig. 1. Diameters of the left ventricular outflow tract (LVOT) were measured in the parasternal left ventricular long axis view during the early phase of ventricular systole. In routine practice, LVOT velocity–time integrals (VTIs) could be acquired in the apical five chamber view or the apical long axis view. In this study we adopted the apical long axis view so that the aorta can also be displayed to ensure the position of doppler sample. LVOT VTI was automatically measured for three consecutive beats and averaged. Cardiac output was calculated using the LVOT diameter, LVOT VTI, and heart rate (HR) in line with the previously mentioned guidelines [8].

**Fig. 2:**
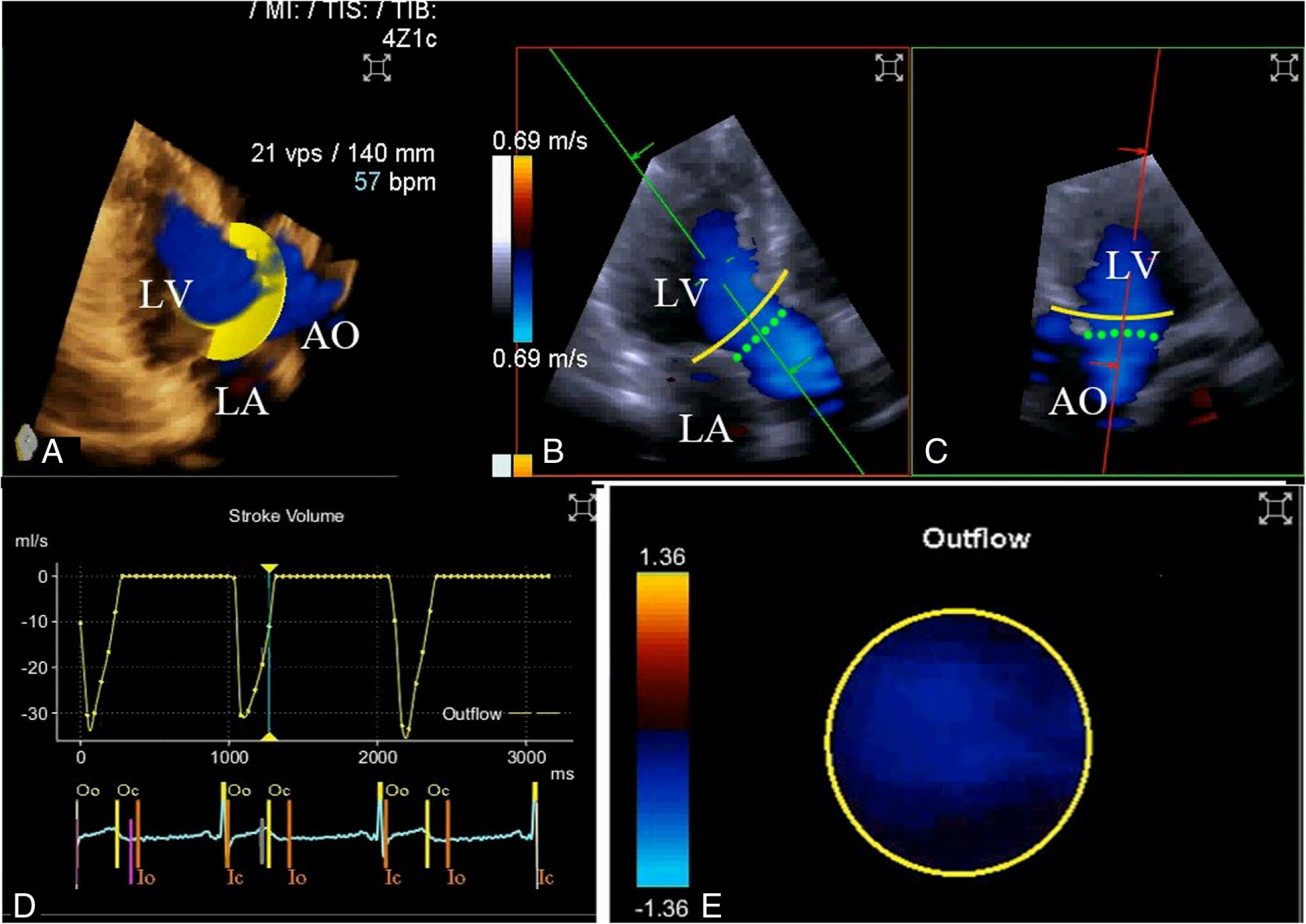
CO assessment using 3DCF transthoracic echocardiography. 3DCF images were obtained in the apical long axis view (panel **a**). With the aortic valve (AV) at the center (green dot line in panel **b** and **c**), the reference images were rotated and adjusted to clearly show the region of interest from the left ventricular outflow tract to the proximal part of the aorta; systole, diastole, timing of AV opening (AVO) and closing (AVC) were automatically recognized by the software according to the ECG (panel **d**); panels **a** and **e** show that the 3D volume color flow passes vertically through the sampling plane (the yellow line in panels **b** and **c**, the yellow plate in panel **a**), and the size of the sampling plane is just big enough for all of the outflow color signals to pass through; panel **d** shows the flow–time curves of three consecutive cardiac cycles generated by the software

3D color flow Doppler images were acquired using the Siemens Acuson SC2000™ Prime ultrasound system with a 4Z1c (1.5–3.5 MHz) volume imaging transducer, as shown in Fig. 2. At least two sets of three consecutive cardiac cycles were obtained in the apical long axis view. The color Doppler scale was adjusted to avoid aliasing. The depth and color Doppler region of interest were optimized to maximize the color volume rates while ensuring that the complete LVOT as well as a part of the aortic root were included. During acquisition, two reference planes were simultaneously displayed at the same time. Digital images were recorded and analyzed using validated offline software (Siemens Medical Solutions) as follows: (1) with the aortic valve (AV) at the center, the reference images were rotated and adjusted to clearly show the region of interest from the LVOT to the proximal part of the aorta; (2) systole, diastole, and the timing of AV opening (AVO) and closing (AVC) were automatically recognized by the software according to the ECG; the timing of AVO and AVC can be manually adjusted by reviewing the loops frame by frame; (3) during systole a frame was selected when the LVOT was fully filled with a color Doppler signal, and the software then automatically placed a hemisphere sampling plane in the LVO; (4) the angle of the sampling plane in the LVOT was manually adjusted so that the 3D volume color flow passed vertically through it and the size of the sampling plane was adjusted according to the cross-section of the 3D volume color flow so that it was just big enough for all outflow color signals to pass through; (5) the sampling plane automatically tracked the plane of interest in the left ventricular outflow tract throughout the whole cardiac cycle; and (6) the software generated flow–time curves of three consecutive cardiac cycles, and then calculated LVOT stroke volume and CO according to the area under the curves.

The echocardiograph experts were blinded to the hemodynamic variables recorded by the FloTrac/Vigileo™ system (these were collected by the intensivist). Systolic, diastolic, and mean arterial pressures and HR were recorded by the real-time bedside monitor. Central venous pressure was recorded through the jugular vein catheter.

### Data collection

The following data were recorded for each patient: (1) baseline demographic data (age, gender, and body mass index); (2) patient’s degree of disease severity (Acute Physiology and Chronic Health Evaluation II [APACHE II] score and European system for cardiac operative risk evaluation [EuroSCORE]); (3) type of surgery (mitral/tricuspid valve replacement, off-pump coronary artery bypass graft [OPCABG], cardiac tumor resection, repair of atrial septal defect); (4) blood gas parameters before enrollment (pH, pCO_2_, HCO_3_^−^, pO_2_, lactate, and hemoglobulin); (5) preoperative echocardiographic parameters (left ventricular ejection fraction [LVEF]); and (6) hemodynamic data at baseline (T0, in a supine position with the head elevated to 30°), after volume loading (T1, 250 mL 0.9% NaCl solution infused in 10 min) and after dobutamine testing (T2, 2 μg/kg/min dobutamine infusion for about 15 min). The hemodynamic data collected in this study included: HR, systolic blood pressure (SBP), diastolic blood pressure (DBP), mean arterial pressure (MAP), central venous pressure (CVP), cardiac index (CI), stroke volume (SV), stroke volume variation (SVV), and CO. (7) echocardiography related data at different time points: CO, SV, VTI and LVEF.

### Statistical methods

Normality was assessed using the Kolmogorov-Smirnov test for all data. Continuous variables are given as means ± SD while categorical variables are reported as adjusted proportions. Comparisons of hemodynamic variables (continuous variables) between different time points were performed by paired Student’s *t*-test or paired Wilcoxon’s test, as appropriate. Differences between categorical variables were compared using the chi-square test or Fisher’s exact test when necessary. Cardiac outputs collected from echocardiography (PWD and 3DCF) were each compared with that from FloTrac using a correlation analysis and by calculating Pearson’s coefficient, expressed as the r value. Agreements of CO measured by the different methods were determined by Bland-Altman analysis, with assessment of the bias as the mean difference between methods. The limits of agreement (LoA) were calculated as the mean ± 1.96 SD from the bias, defining the range in which 95% of the values were expected to fall [17]. The percentage error (PE) was calculated as 1.96 SD of the bias of the methods divided by the mean CO from the two methods, as proposed by Critchley and Critchley, and the two methods could be used interchangeably if the PE was less than 30% [18]. Trending ability was assessed by analyzing ΔCO values on a 4-quadrant plot with concordance. Exclusion zones of 15% were used, as ΔCO data with smaller changes represent a statistical noise component, and a concordance rate of > 92% indicates good trending according to Critchley and colleagues [19]. For all tests, a *p* value < 0.05 was considered statistically significant. Statistical analyses were performed using SPSS 22.0 software (IBM Corporation, NY, USA), GraphPad Prism 5.0, and MedCalc 15.6.1.0 software (Mariakerke, Belgium).

## Results

### Demographic and clinical characteristics of enrolled patients

Demographic and clinical characteristics were summarized in Table 1. Of the 20 post-cardiac surgical patients (seven males, 13 females) enrolled in this study, only three needed low doses of norepinephrine (< 0.05 μg/kg/min) to maintain their blood pressure. A total of 59 pairs of CO measurements were collected between the different methods (one patient’s drainage increased after the infusion of dobutamine). The mean age of the enrolled patients was 62 ± 9 years, and the mean body mass index was 24 ± 4 kg/m^2^. The mean APACHE II score was 7 ± 2, while the mean EuroSCORE was 4 ± 2. The mean LVEF before surgery was 61% ± 10%. The most common surgical treatment in this study was mitral/tricuspid valve surgery (12 patients, 60%); other surgical treatments included coronary artery bypass graft (four patients, 20%), resection of a cardiac tumor (three patients, 15%), and repair of an atrial septal defect (one patient, 5%). All patients were in sinus rhythm and had no severe metabolic abnormalities that had an influence on blood pressure or hemorrhage after surgery.

**Table 1:** Baseline characteristics of the patients (*n* = 20)

Characteristic	Value
Patient	
Age (years)	62 ± 9
Male sex, *n* (%)	7 (35)
BMI (kg/m2)	24 ± 4
EuroSCORE	4 ± 2
APACHE II	7 ± 2
LVEF (%)	61 ± 10
Cardiac surgery category, *n* (%)	
OPCABG	4 (20)
Tumor	3 (15)
ASD	1 (5)
Mitral/tricuspid valve	12 (60)
Blood gas	
PH	7.41 ± 0.04
PCO_2_(mmHg)	36.0 ± 3.9
HCO_3_^−^(mmHg)	22.0 ± 1.7
PO_2_(mmHg)	180.2 ± 54.6
Lactate (mmol/L)	2.0 ± 1.1
Hb(g/dL)	9.9 ± 1.9

Abbreviations: *APACHE* Acute Physiology and Chronic Health Evaluation, *ASD* atrial septal defect, *BMI* body mass index, *CABG* coronary artery bypass graft, *EuroSCORE* European system for cardiac operative risk evaluation, *Hb* hemoglobulin, *LVEF* left ventricular ejection fraction

### Hemodynamic data and echocardiograph related parameters before and after intervention

Hemodynamic parameters at different time points were summarized in Table 2. CO, CI, SV, and SVV were recorded using the FT/V system. There were no significant changes in SBP, DBP, MAP, CO, CI, or SVV after volume expansion; however, SV and CVP increased while HR decreased after volume expansion. After 15 min of dobutamine testing, HR, SBP, DBP, MAP, CO, CI, and SV all significantly increased, while CVP and SVV did not change significantly. At each time point, echocardiography related parameters were collected and the results were shown in Table 3.

**Table 2:** Hemodynamic data (*n* = 20)

	T0	T1	T2
HR (beats/min)	71 ± 12	68 ± 11^a^	79 ± 15^b,c^
SBP (mmHg)	111 ± 15	113 ± 18	121 ± 21^b,c^
DBP (mmHg)	57 ± 8	58 ± 8	65 ± 11^b,c^
MAP (mmHg)	74 ± 11	76 ± 11	87 ± 14^b,c^
CVP (mmHg)	10 ± 3	11 ± 3^a^	11 ± 3
CO (L/min)	3.9 ± 1.0	4.0 ± 0.7	5.1 ± 1.6^b,c^
CI (L/min/m2)	2.4 ± 0.5	2.4 ± 0.4	3.1 ± 0.8^b,c^
SV (ml)	55 ± 13	59 ± 12^a^	64 ± 14^c^
SVV (%)	10 ± 4	9 ± 5	8 ± 4

^a^*p* < 0.05 between T0 and T1; ^b^*p* < 0.05 between T1 and T2; ^c^*p* < 0.05 between T2 and T0. *CI* cardiac index, *CO* cardiac output, *CVP* central venous pressure, *DBP* diastolic blood pressure, *HR* heart rate, *MAP* mean arterial pressure, *SBP* systolic blood pressure, *SV* stroke volume, *SVV* stoke volume variation

**Table 3:** Echocardiography related data (*n* = 20)

Characteristics	T0	T1	T2
3DCF			
CO (L/min)	4.05 ± 0.88	4.23 ± 0.86	5.22 ± 1.8
SV (ml)	55 ± 14	58 ± 12	63 ± 16
2D-PWD			
CO (L/min)	4.63 ± 1.37	4.81 ± 1.50	5.23 ± 1.38
SV (ml)	65 ± 21	70 ± 22	68 ± 23
VTI (cm)	19.02 ± 5.43	20.85 ± 6.15	20.66 ± 6.09
LVEF (%)	62 ± 10	61 ± 10	62 ± 11

Abbreviations: *3DCF* 3 dimensional color flow, *CO* cardiac output, *SV* stroke volume, *2D-PWD* 2 dimensional pulse wave doppler, *VTI* velocity time integral, *LVEF* left ventricular ejection fraction

### Accuracy of CO-PWD and CO-3DCF, compared with CO-FT/V

**Fig. 3:**
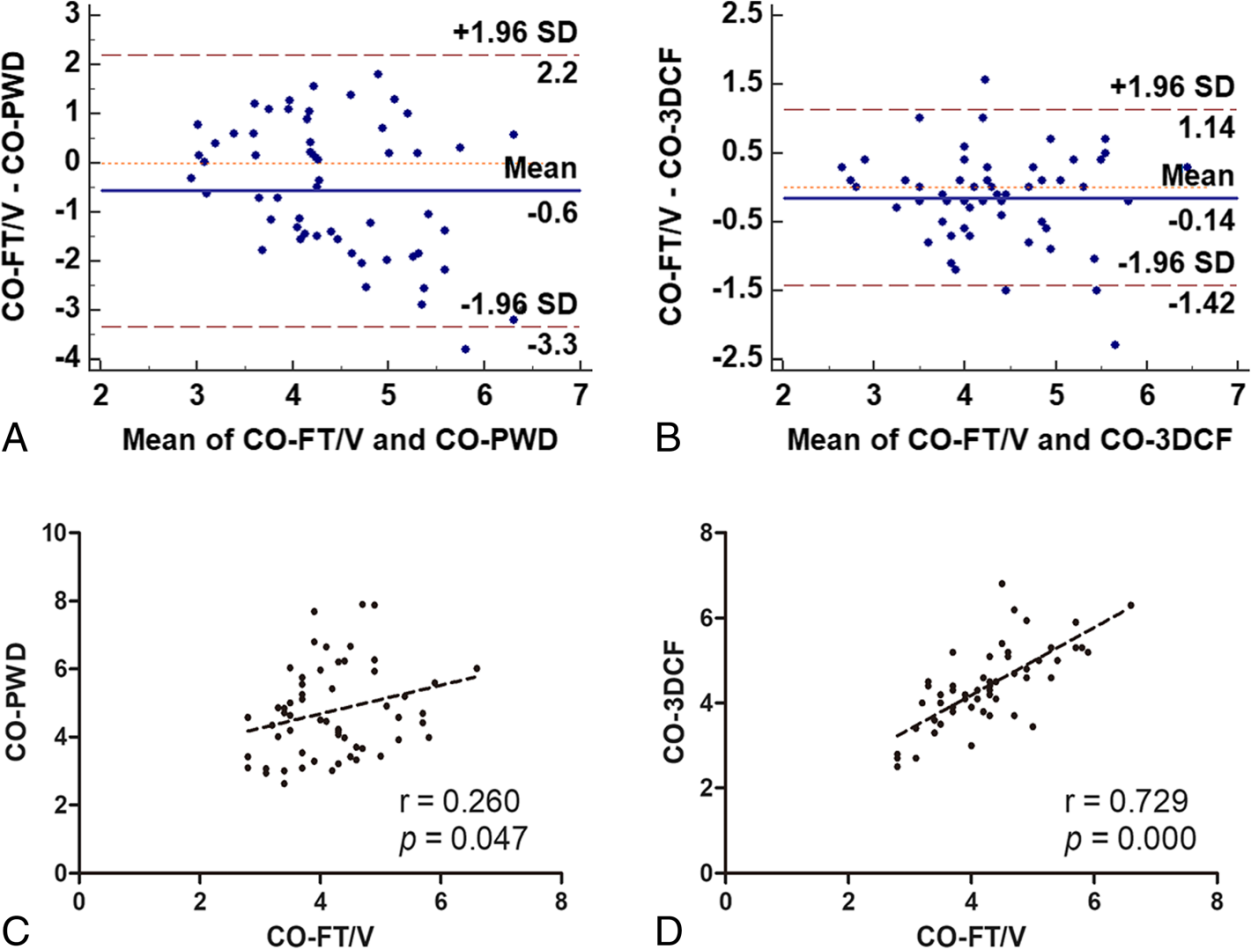
Bland and Altman plot (**a** and **b**) and linear regression plot with Pearson’s coefficient (**c** and **d**) of the cardiac output measured with FT/V versus 2D-PWD and 3DCF echocardiography techniques

Considering all pairs of measurements performed in this study (*n* = 59), Pearson’s coefficients were 0.260 between CO-FT/V and CO-PWD and 0.729 between CO-FT/V and CO-3DCF. Bland-Altman analysis showed the bias between the absolute values of CO-FT/V and CO-PWD was − 0.6 L/min with limits of agreement between − 3.3 L/min and 2.2 L/min, with a PE of 61.3%. The bias between CO-FT/V and CO-3DCF was − 0.14 L/min with limits of agreement between − 1.42 L /min and 1.14 L/min, with a PE of 29.9%. The comparisons between CO-FT/V and CO-PWD, CO-FT/V and CO-3DCF are summarized in Fig. 3.

### Tracking of the trending ability of CO-PWD

**Fig. 4:**
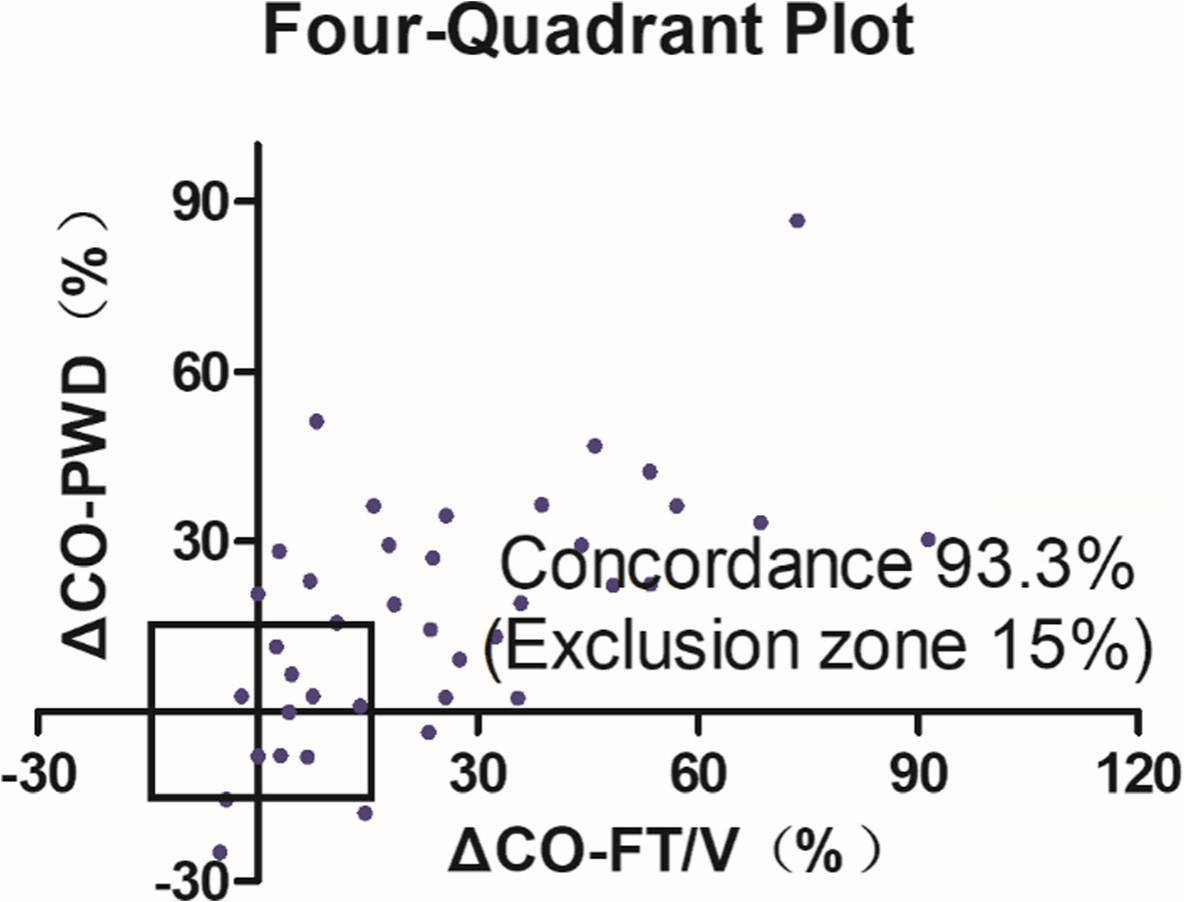
Trending ability of the 2D-PWD echocardiography technique against CO measured by the FT/V system based on four-quadrant concordance analysis

The results of the four quadrant-plot analysis are shown in Fig. 4. As the paired Student’s *t*-test showed no significant difference in CO between T0 and T1, we did not include the ΔCO data before and after volume expansion in the analysis. Among the 39 pairs of ΔCO data induced by the dobutamine test or volume expansion + dobutamine test (T0 to T2 and T1 to T2), 30 pairs exhibited a more than 15% change. The concordance rate between ΔCO-PWD and ΔCO-3DCF was 93.3%.

## Discussion

In this prospective study evaluating the reliability of CO measured by 3DCF and 2D-PWD transthoracic echocardiography in post-cardiac surgical, hemodynamically stable patients, we found that CO values measured by 3DCF echocardiography and FT/V could be considered interchangeable with a PE of 29.9%. Although the precision of 2D-PWD echocardiography in measuring CO was not as good as that of the 3DCF technique, its ability to track changes in CO in different clinical settings was reliable. To the best of our knowledge, this is the first study to evaluate the accuracy of the 3DCF technique in measuring CO after cardiac surgery.

Hemodynamic instability is very common in the early period after cardiac surgery, either because of preoperative status or intraoperative events such as cardiopulmonary bypass [20, 21]. Today, the transthoracic Doppler technique is frequently used because of its convenience, real time measurement, and noninvasiveness [22, 23]. Beside these factors, the accuracy of hemodynamic monitoring is one of the most important concerns of physicians. However, studies concerning the accuracy of 3DCF echocardiography in CO measurement after cardiac surgery have been limited. Due to these considerations, the authors decided to evaluate the accuracy of 3DCF and 2D-PWD transthoracic echocardiography.

In this study, the comparison between the absolute values of CO-FT/V and CO-PWD showed a low bias, but with an unacceptably high LoA and PE (61.3%). This may be due to the inherent limitations of the pulse wave Doppler technique [24, 25]. Firstly, the Doppler technique is angle-dependent and involves the assumption of a uniform flow profile within the LVOT. Secondly, the measurement of the diameter of the LVOT and the assumption of its cross-sectional morphology further reduce the accuracy of the measurement. Mik et al. systemically reviewed studies of CO measurements by echocardiography vs. thermodilution and found that most studies exhibited a small bias, wide LoA, and high PE between the two techniques, but the echocardiography technique was reliable in tracking directional changes in CO. [10] In our study, the four-quadrant plot analysis revealed good concordance (93.3%) between ΔCO-PWD and ΔCO-FT/V, further confirming the ability to track directional changes in CO using the 2D-PWD echocardiography technique.

The percentage error between CO-3DCF and CO-FT/V was 29.9%, which meant that the two methods could be used interchangeably according to Critchley et al. [18] CO was slightly underestimated by the 3DCF technique, with a mean bias of − 0.14 L/min and LoA between − 1.42 L/min and 1.14 L/min. Shimada et al. studied 46 different hemodynamic states in five open-chest pigs to explore the feasibility and accuracy of 2D and 3D color flow Doppler quantification for LVOT SV. SV derived from sonomicrometry was used as the reference value. In their study, the 3DCF method showed a smaller bias and better correlation with the sonomicrometry method than the 2D method [26]. Thavendiranathan et al. also reported real-time volume color flow Doppler is superior to 2D transthoracic echocardiography for the measurement of LV SV using cardiac magnetic resonance imaging as the standard method [27]. 2D techniques involve a multistep process with multiple assumptions: firstly, the small errors in measurements of LVOT are squared in the calculation of LVOT area; secondly, the geometric variation of the LVOT cross-sectional area is neglected; thirdly, the LVOT diameter and its flow velocity are acquired in two different views of two different cycles; and finally, flow velocities vary within the LVOT cross-sectional area. Gated 3D echocardiography eliminates the velocity and geometric assumptions of the 2D method; however, it requires reconstruction of datasets over seven to 14 cardiac cycles. Thus, flow quantification does not truly represent a single beat. Among the three methods, the 3DCF technique not only addresses the above limitations of 2D echocardiography, but also eliminates the need for reconstruction required under gated 3D echocardiography. It can display full volume, B mode, and color Doppler for each single cardiac cycle with a pyramid up to 90° × 90°, and quantify regurgitation, cardiac function, and shunt [27, 28].

There were several limitations of this study. First, its sample size was small, and patients were enrolled because of their good acoustic windows. But not all post-cardiac surgical patients have good acoustic windows, which limits the usefulness of the conclusions in post-cardiac surgical patients. Second, we used a FloTrac/Vigileo™ (third generation) system as the reference method, not the gold standard thermodilution method. Although the patients enrolled were all in good condition and CO measured by the FT/V technique should be accurate in these patients as discussed before, there might still be some discrepancy from the true values. Third, the patients enrolled in this study only represented a subset of ICU patients after cardiac surgery, whether the 3D echocardiography technique adopted would have the same accuracy in all cardiac surgical patients needs further confirmation. Last, this was a single-center study and is subject to intrinsic limitations; a further multicenter study is needed to confirm our findings.

## Conclusions

3DCF transthoracic echocardiography could accurately estimate CO in post-cardiac surgical patients compared with a FT/V system; the two methods could be considered interchangeable. Although 2D-PWD echocardiography was not as accurate as the 3DCF technique, its ability to track directional changes was reliable.

## Abbreviations

2D-PWD: Two-dimensional pulse wave Doppler
3DCF: Three-dimensional color flow Doppler
APACHE II: Acute Physiology and Chronic Health Evaluation II
AV: Aortic valve
AVC: AV closing
AVO: AV opening
CABG: Coronary artery bypass graft
CI: Cardiac index
CO: Cardiac output
CVP: Central venous pressure
DBP: Diastolic blood pressure
EuroSCORE: European system for cardiac operative risk evaluation
FT/V: FloTrac/Vigileo™
HR: Heart rate
LoA: Limits of agreement
LVEF: Left ventricular ejection fraction
LVOT: Left ventricular outflow tract
MAP: Mean arterial pressure
PE: Percentage error
SBP: Systolic blood pressure
SV: Stroke volume
SVV: Stroke volume variation
VTIs: Velocity–time integrals
